# Adherence Connection for Counseling, Education, and Support: Research Protocol for a Proof-of-Concept Study

**DOI:** 10.2196/12543

**Published:** 2019-03-28

**Authors:** Ann-Margaret Dunn Navarra, Marya Viorst Gwadz, Suzanne Bakken, Robin Whittemore, Charles M Cleland, Gail D'Eramo Melkus

**Affiliations:** 1 Rory Meyers College of Nursing New York University New York, NY United States; 2 Silver School of Social Work New York University New York, NY United States; 3 School of Nursing Columbia University New York, NY United States; 4 School of Nursing Yale University New Haven, CT United States

**Keywords:** HIV, smartphone, cell phone, technology, treatment adherence and compliance, methods

## Abstract

**Background:**

The highest rates of new HIV infections are observed in African Americans and Hispanics/Latinos (ethnic minority) adolescents and young adults (youth). HIV-infected ethnic minority youth are less likely to initiate and maintain adherence to antiretroviral treatment (ART) and medical care, as compared with their adult counterparts.

**Objective:**

The objective of this research protocol was to describe our proposed methods for testing a peer-led mobile health cognitive behavioral intervention, delivered via remote videoconferencing and smartphones with HIV-infected ethnic minority youth, Adherence Connection for Counseling, Education, and Support (ACCESS). Our secondary aim was to obtain initial estimates of the biobehavioral impact of ACCESS on HIV virologic outcomes and self-reported ART adherence, beliefs and knowledge about ART treatment, adherence self-efficacy, and health care utilization (retention in care).

**Methods:**

An exploratory, sequential mixed-methods study design will be used with conceptual determinants of adherence behavior informed by the information-motivation-behavioral skills model. HIV-infected ethnic minority youth aged 16 to 29 years with a detectable HIV serum viral load of more than 200 copies/ml (N=25) will be recruited. Qualitative pretesting will be conducted, including semistructured, in-depth, individual interviews with a convenience sample meeting the study inclusion criteria. Preliminary analysis of qualitative data will be used to inform and tailor the ACCESS intervention. Testing and implementation will include a one-group pre-posttest pilot, delivered by a trained *successful* peer health coach who lives with HIV and is well-engaged in HIV care and taking ART. A total of 5 peer-led remote videoconferencing sessions will be delivered using study-funded smartphones and targeting adherence information (HIV knowledge), motivation (beliefs and perceptions), and behavioral skills (self-efficacy). Participant satisfaction will be assessed with poststudy focus groups and quantitative survey methodology. Bivariate analyses will be computed to compare pre- and postintervention changes in HIV biomarkers, self-reported ART adherence, beliefs and knowledge about ART, adherence self-efficacy, and retention in care.

**Results:**

As of December 2018, we are in the data analysis phase of this pilot and anticipate completion with dissemination of final study findings by spring/summer 2019. The major outcomes will include intervention feasibility, acceptability, and preliminary evidence of impact on serum HIV RNA quantitative viral load (primary adherence outcome variable). Self-reported ART adherence and retention in care will be assessed as secondary outcomes. Findings from the qualitative pretesting will contribute to an improved understanding of adherence behavior.

**Conclusions:**

Should the ACCESS intervention prove feasible and acceptable, this research protocol will contribute to a shift in existent HIV research paradigms by offering a blueprint for technology-enabled peer-led interventions and models.

**International Registered Report Identifier (IRRID):**

DERR1-10.2196/12543

## Introduction

The estimated prevalence of HIV-infection in the United States was 1.1 million among persons aged 13 years and older at year-end in 2015 [1]. More recently, in 2016, new HIV diagnoses in the Unites States totaled 39,782 with 41% represented by adolescents and young adults (youth) aged 15 to 29 years [2]. African Americans and Hispanics/Latinos (ethnic minority) youth are disproportionately affected by HIV infection with epidemiologic data highlighting sexual contact as the most common transmission category [3]. Initiation of antiretroviral treatment (ART) is recommended for all HIV-infected individuals [4]. Maintenance of optimal adherence to ART, defined as more than 95% for unboosted protease inhibitor–containing regimens and 80% adherence for boosted protease inhibitor regimens [5,6] is the single most important criterion to prevent ART and virologic failure [7,8], HIV-related morbidity and mortality [4,9], and behavioral transmission to seronegative individuals [4,10]. Among HIV-infected ethnic minority youth, suboptimal adherence is highly prevalent [11-17] with resulting HIV RNA viral suppression estimates of 30.5% (577/1891) [18] to less than 6.0% (4449/78,949) [19], thereby significantly increasing risk of sexual transmission [10,20-22]. HIV-infected youth are also less likely to initiate ART and be retained in care [19] with estimates of 56% (17,874/32,149) maintained in continuous HIV care during 2015 [23].

Both psychosocial and structural factors have been implicated as barriers to ART adherence with structural factors including travel to health care settings [24,25] and stigma [24,26]. Among psychosocial barriers to adherence are beliefs of ART futility [27] and concerns about use [27,28], ART knowledge and understanding [29], trauma [30-32], substance use and psychological distress [5], and decreased self-efficacy [33,34]. Facilitators of ART adherence include information and communication technologies [35], HIV/ART education [4,29,36], beliefs of medical necessity [25,28] and positive outcome expectancy of ART [37], and higher levels of self-efficacy [34]. Moreover, self-efficacy has been shown to mediate the relationship between ART adherence and stigma among HIV-infected adults [38].

To date, evidence from systematic and integrative reviews demonstrate a paucity of effective targeted adherence interventions for HIV-infected ethnic minority youth [15,39-45]. Cognitive behavioral interventions (CBIs) have shown promise to improve adherence in HIV-infected adults [46-48] and youth [49], and are designed to reduce cognitive biases (negative thoughts and beliefs) and build effective adherence self-management skills [50]. CBIs may be delivered using motivational interviewing (MI) techniques including expressing empathy and forming collaborative partnerships with participants [46]. Mobile media platforms represent a viable option to deliver behavioral interventions targeting ART adherence [36,51-53] and offer the potential to mitigate structural barriers, including travel to health care settings [54]. When used for delivery of behavioral interventions, these platforms allow for improved confidentiality, thereby decreasing fears related to anticipated HIV-related stigma [26] or inadvertent disclosure of HIV seropositive status during an HIV clinical encounter visit.

Improved health outcomes for HIV-infected ethnic minority youth are dependent on the design and implementation of interventions that entail community mobilization [55], such as peer support and counseling [24,56,57]. In addition to sharing experiential information, there is evidence to demonstrate that peers provide emotional support by conveying understanding and acceptance, thereby allowing for discussion of negative emotions [58]. Research findings show that peers competently deliver HIV behavioral interventions targeting ART adherence [57] and retention in care [59] and contribute to improved adherence [57] and retention outcomes [60].

In our pilot research including a cross-sectional descriptive survey design, beliefs of positive outcome expectancy were associated with optimal self-reported adherence to ART [37], and in a related substudy, ownership of cellular phones with internet access was commonly reported among HIV-infected ethnic minority youth [37,61]. In fact, the cell phone was the preferred route to communicate with a health care provider [37,61], a finding that is congruent with the observed increased patterns of smartphone ownership among ethnic minority youth in the United States [62,63]. Evidence from one small pilot demonstrated that remote videoconferencing is feasible and acceptable for adherence counseling, when delivered to HIV-infected ethnic minority youth on site, within the clinical setting. However, it has not yet been tested for preliminary efficacy in the community setting [64]. Given the high rates of suboptimal adherence to ART and poor retention in care among HIV-infected ethnic minority youth, there is a pressing need to develop and test novel interventions**.** Therefore, in this research protocol, we present the methods for an innovative proof-of-concept study, Adherence Connection for Counseling, Education, and Support (ACCESS). We expect that implementing this peer-led mobile health (mHealth) CBI delivered via remote videoconferencing using smartphones will be feasible and acceptable with the potential to influence ART adherence in HIV-infected ethnic minority youth.

## Methods

### Objectives

The primary aim of the ACCESS proof-of-concept study is to characterize the feasibility and acceptability of a peer-led mHealth CBI delivered via remote videoconferencing using smartphones. Our secondary aim is to obtain initial estimates of the biobehavioral impact of ACCESS on HIV virologic outcomes and self-reported ART adherence, beliefs and knowledge about ART treatment, adherence self-efficacy, and health care utilization (retention in care). The major outcomes of this pilot study will include intervention feasibility, acceptability, and preliminary evidence of impact with respect to biobehavioral outcomes, namely, serum HIV RNA quantitative viral load (primary adherence outcome variable). Self-reported ART adherence and retention in care will be assessed as secondary outcomes.

### Theoretical Foundation

**Figure 1:**
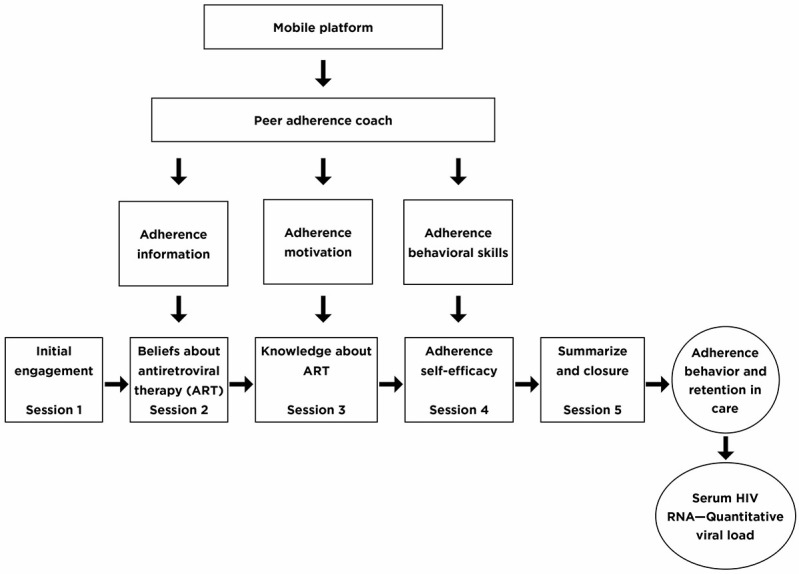
The potential impact of the Adherence Connection for Counseling, Education, and Support intervention on adherence behavior.

The information-motivation-behavioral (IMB) skills model of antiretroviral adherence [65] will be used to identify the conceptual determinants of adherence behavior, namely, adherence information, motivation, and behavioral skills. These determinants are operationalized as knowledge and beliefs about ART and adherence self-efficacy. The primary adherence outcome variable is serum HIV RNA quantitative viral load. Recently, conceptualization of treatment adherence has been broadened to include linkage and retention in care [4] and, therefore, the IMB model was modified to include retention in care as an outcome variable (Figure 1).

During remote videoconferencing sessions, peer-health coaches will use cognitive behavioral strategies delivered using MI techniques to enhance problem solving and target beliefs and knowledge about ART and adherence self-efficacy for improved adherence behavior (Figure 1). The IMB model is supported for use with technology-enabled adherence interventions [66,67] and CBIs [68,69] delivered using MI techniques [70]. An assumption of this model is that motivation to adhere has a social component which is influenced by perceived social support received from important others including health care workers [65]. This supports the inclusion of peers [57,71]. Given the high prevalence of past traumatic [32] and stigmatizing [72] experiences among HIV-infected individuals, these constructs will be included in the approach as potential mediators or moderators of study outcomes.

### Study Design

**Figure 2:**
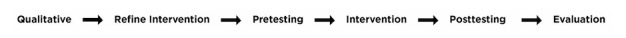
Schematic design of the Adherence Connection for Counseling, Education, and Support proof-of-concept study.

An exploratory, sequential mixed-methods study design will be used (Figure 2) in a sample of HIV-infected ethnic minority youth (aged 16 to 29 years). Qualitative pretesting will be conducted with a convenience sample of HIV-infected ethnic minority youth meeting the study inclusion criteria. A preliminary analysis of qualitative data will be used to inform and tailor the ACCESS intervention. Testing and implementation of the ACCESS intervention will include a one group pre-posttest pilot with delivery by an HIV-infected trained peer-health coach. The sample size for the pilot will be 25 HIV-infected ethnic minority youth.

### Participants and Recruitment

After receiving approval from designated institutional review boards, recruitment of HIV-infected ethnic minority youth will be initiated from 3 urban HIV centers in New York City, 2 public hospital centers, and 1 HIV nonprofit community health plan providing health care coverage to chronically ill Medicaid recipients. Recruitment efforts, effective with our previous work [37], will include posting of flyers and regularly scheduled visits to the outpatient clinical settings during times of high patient volume.

Inclusion criteria will include the following: HIV seropositive status (behaviorally and perinatally infected youth), aged 16 to 29 years, English speaking, current ART with a prescribed regimen, detectable quantitative HIV serum viral load more than 200 copies/ml, and no neurocognitive deficits which would impede participation in videoconferencing sessions or completion of study measures. Screening with the Folstein Mini-Mental State Exam (MMSE) will be performed to assess for the presence of neurocognitive deficits [73]. Participants with a score of 24 or greater will be eligible for study participation and this cutoff score is on the basis of our prior experience [37] and findings from other published evidence with HIV-infected ethnic minority youth [74].

### Qualitative Pretesting

A convenience sample will be recruited. We will obtain informed consent and assent for 16- and 17-year-old participants before beginning qualitative data collection. Participants will meet study inclusion criteria serving as informants to the ACCESS intervention. Yet, these participants will also be eligible to participate in the ACCESS intervention. The estimated sample size is 12 to 15 participants; however, new participants will be interviewed until data saturation is reached [75]. The IMB skills model will be applied for the development of the interview guide. In-depth, individual, semistructured interviews including open-ended questions with probes will be conducted to gather qualitative data on the perceptions and beliefs of HIV-infected ethnic minority youth regarding the proposed study design, barriers and facilitators of ART adherence, and potential impact of the ACCESS intervention on ART adherence and retention in care. Interviews will be conducted in a private setting at each of the clinical agencies. Reflective notes on the events and processes observed during the interviews will be maintained, in addition to recording emerging codes, themes, and or any concerns [76]. All interviews with study participants will be digitally recorded, transcribed verbatim, and analyzed using ATLAS.ti (v8.2 Microsoft Windows), a software program for organizing, coding, and analyzing qualitative data. Directed content analysis will be conducted [77] to generate categories and broad themes, informing the ACCESS intervention. Methodological rigor will be ensured by performing member checks, establishing an audit trail, collecting thick descriptive data, and triangulating data sources [78].

### Study Procedures

Upon obtainment of informed consent, and assent for 16- and 17-year-old participants, data collection will begin with administration of the Folstein MMSE [73] by the principal investigator (PI) or research assistant (RA) to screen for presence of neurocognitive deficits. Baseline virologic adherence estimates will be collected preintervention and postintervention including weeks 8, 16, and 24. The rationale for selection of these time points is because viral suppression (indicated by HIV RNA less than 48 copies/ml) could be expected to occur in 8 to 24 weeks among participants adhering to ART and without evidence of phenotypic or genotypic resistance to ART [4]. Self-reported adherence, beliefs and knowledge about ART, adherence self-efficacy, and retention in care will be measured at pre- (baseline) and postintervention. Survey instruments (described in the Measures section) will be completed by the participant on-site in the presence of the PI or trained RA (Table 1).

**Table 1:** Plan for data collection of primary study variables.

Data source	Baseline, preintervention	Post ACCESS^a^ intervention	8 weeks postintervention	16 weeks postintervention	24 weeks postintervention
Self-reported adherence	X^b^	X	—^c^	—	—
Serum HIV RNA viral load	X	X	X	X	X
Beliefs	X	X	—	—	—
Knowledge	X	X	—	—	—
Self-efficacy	X	X	—	—	—
Health care utilization (retention in care)	X	—	—	—	X

^a^ACCESS: Adherence Connection for Counseling, Education, and Support.

^b^X: represents a data collection time point.

^c^—: represents a time point at which data was not collected.

### Allocation of Smartphones

Participants will be provided with a study-funded smartphone to allow for uniform and uninterrupted access to the intervention. To minimize the total number of smartphones needed, the smartphones will be returned after completion of the intervention phase, reset to factory settings by university information technology (IT) staff, and reassigned to new enrollees. Return and reset of research-funded smartphones provided for HIV-infected minority youth has been demonstrated within the study procedures for a technology-supported behavioral intervention [79].

### Mobile Platform

In conjunction with the university IT administrative staff, WebEx Communications Inc will be used to provide a secure and Health Insurance Portability and Accountability Act–compliant media platform for implementation of the remote videoconferencing sessions. WebEx mobile apps will be downloaded by IT staff to study-funded smartphones allocated to participants before initiation of videoconferencing sessions. Each study participant will be trained on the use of WebEx mobile apps by the PI upon receipt of their study phone. This education will also detail the necessity for receiving remote videoconferencing sessions in a private location using their password-protected study-funded smartphone. To deliver the intervention, the trained peer health coach will access WebEx remote videoconferencing sessions from a university-designated computer located in a private office within the academic setting. Members of the study team will be present and available to support the adherence coach during these scheduled sessions.

### Training of Peer Health Coaches

A total of 2 *successful* peer health coaches who live with HIV and are well-engaged in HIV care and taking ART will be hired to deliver the ACCESS intervention, after completing a comprehensive training program. The training program will include a minimum of 40 hours of instructions [80] delivered during a 4-month period by content experts in their respective fields (ie, HIV, MI, and IT). The approach for education of peer health coaches will be guided by criteria and select resources from an existing national peer training program for HIV-infected individuals, *Train the Trainer* [81], and the HIV peer development toolkit [82].

The training format will be divided into 3 phases including instructive lectures and discussions, written practice for reinforcement of didactic material, and live role-playing of the intervention protocol using WebEx videoconferencing. Instructive lectures will be directed to impart knowledge on HIV disease, treatment adherence, HIV stigma, trauma, and ethical/human subjects’ considerations, role of the peer and professional boundaries, MI techniques [83], and stages of change [84]. To reinforce MI techniques and other training content, written practice worksheets will be developed and included as homework assignments. Members of the research team will role-play using deidentified data from completed qualitative interviews, allowing peer health coaches to gain familiarity with adherence struggles frequently encountered by the enrolled study population. Ongoing feedback and support from the PI and study team will be offered to peer health coaches at all phases of training.

### Adherence Connection for Counseling, Education, and Support Adherence Intervention

A cognitive behavioral approach delivered using MI techniques will target beliefs and knowledge related to ART, and adherence self-efficacy during the peer-led mobile videoconferencing sessions (ACCESS sessions 1 to 5). Problem-focused coping strategies will be used [46] to help participants manage common adherence challenges. Peer health coaches will also assist participants to define struggles related to medication adherence and brainstorm for possible solutions [46]. Consistent with the spirit of MI, peer health coaches will strive for a partnership with participants; motivation-to-change adherence behavior will be elicited by the participant and not because of direct persuasion [85]. Peer health coaches will recognize that readiness to change is a fluctuating process and that ambivalence is common [85]. Acceptance, affirmation, reflective listening, and freedom of choice will be among the MI tenets used for communication between the peer health coach and participant [85]. During each of the ACCESS sessions, peer health coaches will be attentive to change talk [85,86], and ask permission before delivering any unsolicited HIV health information [86] and or sharing experiences. The open questions, affirmation, reflective listening, and summary reflections skills-based model will be practiced by peer health coaches [83]. A formal, comprehensive written intervention protocol will be used.

At a mutually agreed time, a member of the study team will schedule the participant for each of the 5 weekly 60-min peer-led ACCESS sessions. After initial assignment to 1 of 2 peer health coaches, the participant will *meet* with this peer health coach for all 5 sessions. The rationale for the selection of 5 sessions is on the basis of the best available evidence from technology-delivered interventions using videos [87,88] and videoconferencing with ethnic minority HIV-infected adults [89,90] and psychoeducational interventions using applied technology in youth with type 1 diabetes [91,92]. In the event of a participant canceling or missing a scheduled session, appointments will be rescheduled within the week by the PI or RA. Active supervision of peer health coaches will include regular meetings with the PI and study team and review of audio-video intervention sessions to assess intervention fidelity.

#### Overview of Adherence Connection for Counseling, Education, and Support Sessions 1 to 5

ACCESS Session 1 will serve to engage the participant in the study, establish credibility of the study team, and foster peer-participant partnership [85]. Open-ended questions, affirmation, reflection, and restatement [83] will be used by the peer health coach to gain understanding of the participant’s HIV history and experiences with ART, while sharing their own experiences living with HIV. This session will also allow for initial discussion of the participant’s beliefs and perceptions influencing adherence motivation and allow the participant to establish their agenda and goals for study participation [86].

ACCESS Session 2 will provide continued support for participant engagement. Peer health coaches will continue to explore adherence motivation by eliciting self-perceived barriers and facilitators of adherence [93] and discussion of beliefs surrounding ART [4,25,37]. Participant beliefs conducive to optimal ART adherence (ie, association between nonadherence and illness) will be encouraged and negative beliefs related to ART explored. Support systems will be examined, recognizing that HIV stigma, either perceived or real, leads to social isolation, thereby influencing health behavior [26,94].

ACCESS Sessions 3 will emphasize adherence information and knowledge about ART, as relevant ART adherence information is a prerequisite of consistent use of ART medications [65]. HIV education to support treatment adherence will be offered by viewing a video titled *Understanding HIV: Basics* [95]. Peer health coaches will provide a neutral context for information exchange [86] related to eliciting participant’s understanding of video content presented including HIV health information, treatment adherence, ART side effects, and HIV biomarkers [4,96].

ACCESS Session 4 will offer a forum for dialogue of adherence behavioral skills or objective and perceived abilities (ie, self-efficacy) related to ART adherence *.* Recognizing that increased self-efficacy is associated with ART adherence [34], peer health coaches will facilitate the participant’s identification of strategies to promote and reinforce adherence self-efficacy. During this session, peer health coaches will elicit from the participant behaviors leading to periods of optimal treatment adherence, while sharing their experiences in successfully maintaining adherence. Additionally, stressors, situations, and or events leading to ART missed doses among participants will be explored. Peer health coaches will facilitate active problem solving for the development of more effective adherence self-management skills tailored to the lifestyle needs of the individual participant [4]. The session will conclude with participant goal setting to support HIV health and ART adherence, cognizant of adherence barriers and facilitators discussed in prior sessions.

ACCESS Session 5 will allow for discussion of strategies to facilitate retention in care and provide time for intervention closure including review, reflection, and summary [83] of completed content from past sessions. The participant’s history in adhering to HIV medical appointments (retention in care) will be explored by peer health coaches. Peer health coaches will elicit barriers, while sharing approaches for successfully maintaining HIV medical appointments, in response to challenges identified by participants. Goal setting initiated during ACCESS Session 4 will also be revisited with affirmation of progress reported. If the participant was unable to maintain their designated goal, the peer health coach will acknowledge the struggle and elicit contributing factors. This session will close with the peer health coach thanking the participant for their time, expressing respect for their autonomy and choices related to adherence behavior. An opportunity to provide additional feedback and or ask questions will be provided.

Participants will be compensated with gift cards for their time as follows: US $15 for each completed ACCESS videoconferencing session (US $75 for 5 sessions), and US $25 for each pre- and postintervention data collection visit (US $50 total). Participants completing qualitative interviews will be compensated for their time and travel with an additional US $25 gift card. Therefore, the total compensation for completing all 5 intervention sessions and pre- and postintervention follow-up will be US $125. If participating in the qualitative component, total compensation will be US $150.

#### Measures

Collection of baseline demographic and clinical data will be conducted using a PI-created instrument that includes the following: age, gender, level of education, mode of transmission, staging of HIV disease–AIDS diagnosis, length of time of current ART regimen, diagnosis of depression, and substance use. Depression and substance use are systematically assessed by health care professionals at recruitment sites and documented in the medical record. Medical record data extraction to assess depression and substance use among HIV-infected ethnic minority youth is feasible on the basis of our prior work [37].

Beliefs about ART will be measured with the Beliefs About Medication Scale. This 59-item health beliefs questionnaire uses a 7-point Likert scale to perceived threat, positive and negative outcome expectancy, and intent regarding oral medication adherence. Reliability (Cronbach alpha=.79-.87; test-retest reliability, *r*=.71-.77) and validity have been demonstrated in youth with chronic illness (n=133) [97].

Knowledge about ART will be measured with the HIV Treatment Knowledge Scale. This 21-item instrument uses true and false questions to assess knowledge of adherence, side effects, and antiretroviral resistance. Test-retest reliability (*r*=.83) and internal consistency (CFI>0.90) are satisfactory when tested with HIV-infected adults [98].

Adherence self-efficacy or the sense of being able to adhere to prescribed HIV medications [99] will be measured with the Adherence Self-Efficacy Scale. This 12-item survey measure uses a 10-point scale (0=cannot do it all; 10=completely certain can do it) to assess confidence in ability to carry out important treatment-related behaviors [100]. Psychometric evaluation demonstrates robust internal consistency (*r*=.90) and test-retest reliability when used with HIV-infected adults (*r*>.70).

### Adherence Outcomes

#### Self-Report and HIV Biomarkers

A 3-day self-report of ART adherence will be measured to describe subjective adherence behavior. To minimize the potential for bias while collecting adherence estimates, questions will be worded in a nonjudgmental style assuming missed doses. For example, the PI or RA would ask, “before beginning the questionnaires, could you please tell me how many doses of medicine you missed yesterday?” Using this information, we will compute an average missed dose calculation: number of doses missed per medication multiplied by dosing schedule during the past 3 days divided by total number of prescribed doses over the past 3 days. This percentage will be subtracted from 100% to obtain the 3-day self-reported adherence estimate [101]. This method has demonstrated feasibility, acceptability, and validity in our pilot work with HIV-infected ethnic minority youth [37] and in other studies [102].

Serum HIV RNA quantitative viral load is the primary adherence outcome variable and will be measured to eliminate the potential for social desirability bias associated with subjective adherence reports. HIV viral load is a robust predictor of ART adherence in both HIV-infected ethnic minority youth [101,103,104] and adults [102,105]. Medical record data extraction will be performed to access HIV viral load results.

#### Health Care Utilization (Retention in Care)

A gold standard for measuring retention in care has not been established, and therefore, selection of a retention measure may be tailored to context [106]. For the purposes of this proof-of-concept study, retention in care will be calculated as a proportion of kept to scheduled visits (range 0%-100%); the denominator will exclude canceled visits [106,107]. Retention data for HIV health care visits will be extracted from the medical record and 6-month pre- and postintervention retention estimates compared.

### Feasibility and Acceptability

#### Participant Satisfaction

The Client Satisfaction Questionnaire [108] will be administered at the conclusion of the intervention. This 8-item survey measure has been widely applied and is valid, reliable, and feasible for use with HIV-infected ethnic minority youth participating in technology-supported behavioral interventions [109]. Additionally, during ACCESS Session 5, the trained peer health coach will ask participants to briefly describe what was helpful and/or not helpful about any of the videoconferencing sessions. A trained RA will review and transcribe these videoconferencing segments.

#### Participant Acceptability With the Intervention

At the conclusion of the intervention phase, study participants will be asked to participate in a one-time session 60-min focus group to share feedback on acceptability of the intervention, quality of the video interactions, what was learned, strengths and weakness, and recommendations for improvement. Focus group sessions will be led by the PI and digitally recorded. A series of open-ended questions with probes will be developed to distinguish essential aspects of the information.

#### Intervention Fidelity

All audiotapes of the videoconferencing sessions will be reviewed. Fidelity to the study protocol by the peer health coaches will be assessed by a trained RA.

### Sample Size and Statistical Power

The purpose of this proof-of-concept study is to test the concept of implementing a peer-led mHealth CBI delivered via remote videoconferencing and smartphones. It will not be a definitive test of intervention efficacy, and therefore, a power calculation will not be computed.

### Data Analysis Plan

Data will be imported into SPSS Statistics, version 25 (IBM). Initially, we will compute descriptive statistics (mean and SD, median and range, and frequency and percentage) to summarize the following: psychosocial and demographic characteristics of the study population, adherence estimates, scores on survey instruments (beliefs, knowledge, and self-efficacy), and retention in care. Data will be assessed for normality, and nonparametric statistics will be used for data that are not normally distributed.

To test aim 1 (feasibility and acceptability of ACCESS), descriptive statistics will be computed summarizing participant response rates to remote videoconferencing sessions including the number of missed and rescheduled appointments, recruitment, and overall study retention and attrition rates. To evaluate participant satisfaction with the intervention, scores on the client satisfaction questionnaire [108] will be measured postintervention and summarized. Additionally, a descriptive analysis and summary of transcribed content from ACCESS Session 5 will be performed, including a participant description of what was helpful and not helpful about any of the videoconferencing sessions. All sessions will be appraised for fidelity to the intervention protocol. A subset will be randomly selected and MI skills of the peer health coaches appraised. Qualitative data obtained from postintervention focus groups will be reduced to categories and broad themes. ATLAS.ti (v8.2 Windows) will be used for data analysis.

To test AIM 2 (potential impact of ACCESS), the primary outcome variable of adherence as measured with serum HIV RNA will be dichotomized as a binary variable (less than 200 copies/ml; more than 200 copies/ml), with more than 200 copies/ml indicating virologic failure [4]. All participants will be unsuppressed at baseline. An interval estimate of the proportion with viral suppression at follow-up will indicate the potential impact of ACCESS. Changes in log_10_ viral load, scores of self-efficacy, beliefs about medications, knowledge about ART, self-reported adherence, and retention in care will be compared before and after the ACCESS intervention using McNemar chi-square or exact test for discrete variables, and paired *t* tests for continuous variables. Changes from baseline to postintervention will be explored. Bivariate associations with follow-up viral suppression will be estimated for other variables such as depression, substance use, and viral load at baseline. Multivariate analysis will not be performed, given the modest sample size for the pilot study.

## Results

As of December 2018, we are in the data analysis phase of this pilot and anticipate completion with dissemination of final study findings by spring/summer 2019. Findings will determine the feasibility and acceptability of ACCESS, a peer-led mHealth CBI delivered via remote videoconferencing, using smartphones for HIV-infected ethnic minority youth. We also expect that the study findings will provide preliminary evidence of the potential impact of ACCESS on serum HIV RNA quantitative viral load and self-reported ART adherence and retention in care as secondary outcomes. Results of the qualitative pretesting will contribute to a better understanding of the information, motivation, and behavioral skills associated with ART adherence behavior in this high-risk cohort. Criteria from the Consolidated Standards of Reporting Trials of Electronic and Mobile HEalth Applications and onLine TeleHealth will be used to report final study results [110].

## Discussion

### Overview

We describe the design and methods for a novel peer-led mHealth CBI delivered via remote videoconferencing, using smartphones in HIV-infected ethnic minority youth. The objectives of ACCESS project are consistent with the goals and strategies of the national HIV/AIDS plan calling for increased access to HIV care, improved ART adherence support, and reduced health disparities among high-risk HIV-infected populations including ethnic minority youth [111]. Our approach has multiple strengths including the integration of peer health coaches, technology (videoconferencing and smartphones), and the mixed-methods study design. Therefore, we expect for the findings from this study to make several important contributions. These include establishing intervention feasibility and acceptability and offering preliminary estimates of impact on ART adherence and HIV biomarkers. Additionally, retention in care is a secondary study outcome, and these data will add to a body of evidence in need of development [44,112]. The ACCESS proof-of-concept study is also systematically designed to integrate qualitative data from important stakeholders (HIV-infected ethnic minority youth) [113,114]. Mixed-methods design is recommended when qualitative data will be used to inform the development of an intervention, and also for gaining a more complete understanding of a complex problem [115].

The ACCESS adherence intervention will be delivered by an HIV-infected ethnic minority trained health coach, representing a distinct approach to mitigate perceived stigma and bridge the gaps between the health care system and HIV-infected youth [81,116,117]. Published results of a recent meta-analysis provide evidence for building peer support into health care models for HIV-infected individuals [94]. More specifically, study findings show associations between experiencing HIV-related stigma and lowered levels of social support, ART adherence, and access to health care [94].

The ACCESS intervention is technology-enabled (videoconferencing and smartphones), allowing for a developmentally acceptable mode of communication between study participants and peer health coaches. As clinic-based interventions are limited in scope and scalability, technology is an ideal fit to support behavioral interventions with HIV-infected youth [53,118,119]. Moreover, delivery of technology-enabled interventions for HIV-infected individuals is associated with improvements in ART adherence [120], HIV biomarkers [121], engagement [122], and retention in care [121], while serving to extend reach and improve efficacy of HIV interventions [123].

### Limitations

Although our approach has many strengths, the methodological limitations include a small sample size and potential for social desirability bias with self-reported adherence estimates. However, these estimates will be validated with HIV biomarkers.

### Conclusions

In conclusion, most new cases of HIV-infection are sexually transmitted among ethnic minority youth [3] for whom adherence to ART is a major challenge. Staggering disparities exist in the HIV cascade of care, including the lack of sustained HIV viral suppression and poor linkage to care [19,124]. Presently, there is an urgent need for effective strategies to improve ART adherence among HIV-infected ethnic minority youth [125]. Should the ACCESS adherence intervention demonstrate feasibility and acceptability, this research protocol will offer a blueprint for the development of technology-enabled peer-led interventions and models.

## Abbreviations

ACCESS: Adherence Connection for Counseling, Education, and Support
ART: antiretroviral treatment
CBI: cognitive behavioral intervention
IMB: information-motivation-behavioral
IT: information technology
MI: motivational interviewing
mHealth: mobile health
MMSE: Mini-Mental State Exam
PI: principal investigator
RA: research assistant
